# Common Peroneal Nerve Palsy Due to a Proximal Tibiofibular Joint Ganglion Cyst in a Recreational Runner: A Case Report

**DOI:** 10.7759/cureus.92842

**Published:** 2025-09-21

**Authors:** Luís Amaral Oliveira, Ana Margarida Esteves, Bruno Cancela, David Moura, Rodrigo Correia, Andre Borges, José Luís Carvalho

**Affiliations:** 1 Physical Medicine and Rehabilitation, Centro de Reabilitação do Norte, Unidade Local de Saúde Gaia e Espinho, Porto, PRT; 2 Physical Medicine and Rehabilitation, Centro Hospitalar de Entre Douro e Vouga, Santa Maria da Feira, PRT; 3 Physical Medicine and Rehabilitation, Trofa Saúde Gaia, Porto, PRT

**Keywords:** case report, common peroneal nerve palsy, foot drop, ganglion cyst, proximal tibiofibular joint, recreational runner, ultrasound-guided aspiration

## Abstract

Common peroneal nerve (CPN) palsy is one of the most frequent lower-limb compression neuropathies in athletes, typically occurring at the fibular neck. Proximal tibiofibular joint (PTFJ) ganglion cysts are a well-recognized but rare extraneural cause of CPN compression, especially in runners. We report a 36-year-old female recreational long-distance runner who presented with a six-month history of progressive lateral knee pain that acutely evolved into foot drop. Examination revealed severe weakness in ankle dorsiflexion and eversion (Medical Research Council grade 1/5) and sensory loss over the dorsum of the foot. Electrophysiological studies demonstrated a >50% conduction block of the CPN at the fibular head with slowed velocity and signs of denervation. Magnetic resonance imaging (MRI) showed a large (6 × 1.1 cm), multiloculated cyst arising from the PTFJ and compressing the CPN. The patient opted for conservative management: an ultrasound-guided aspiration of the cyst with corticosteroid injection was performed, followed by a rehabilitation program. This yielded fast improvement; within one month, her foot drop had resolved, and dorsiflexion strength improved significantly. By three months, pain had nearly resolved and gait had normalized, although the cyst recurred on imaging; a second aspiration was done. At six months, motor function remained normal with only mild residual paresthesia, and surgical excision was recommended for definitive treatment. This case highlights that image-guided aspiration combined with rehabilitation can lead to swift and meaningful recovery in CPN palsy despite severe initial neurologic deficits, though cyst recurrence is common if the articular connection is not addressed. PTFJ ganglion cysts, although rare, should be considered in the differential diagnosis of foot drop in athletes.

## Introduction

Common peroneal nerve (CPN) palsy is one of the most frequent compression neuropathies of the lower limb, owing to the nerve’s superficial course around the fibular neck. While traumatic injuries and external compression are well-recognized causes of CPN palsy, non-traumatic etiologies such as cystic lesions of the proximal tibiofibular joint (PTFJ) are rare but important to consider - particularly in athletes.

The first description of a PTFJ ganglion cyst dates back to 1891 [1]. These lesions remain uncommon, with an estimated prevalence of ~0.76% in patients undergoing knee magnetic resonance imaging (MRI) [2,3]. The largest review to date, comprising 242 cases, reported a mean patient age of 41 years, male predominance (71%), and neurological involvement in most symptomatic cases [4]. Although PTFJ cysts have been documented across a wide age range (from early childhood to the elderly), presentation with CPN palsy is unusual and often delayed, increasing the risk of incomplete nerve recovery [5]. Reports in runners are especially rare, with only isolated cases reported in the literature [3,6].

PTFJ ganglion cysts can be classified by location relative to the nerve as intraneural (the most common type, ~39% of cases), extraneural (~26%), combined intra- and extraneural (~15%), or intraosseous (~4%) [4]. Intraneural cysts are thought to result from synovial fluid dissecting along the recurrent articular branch of the CPN into the epineurium, whereas extraneural cysts originate outside the nerve sheath from the PTFJ capsule, causing external compression of the nerve [7]. The anatomical course of the CPN beneath the biceps femoris tendon and around the fibular neck makes it highly susceptible to compression by such lesions [5,8].

Diagnosis is based on clinical examination supported by imaging. Neurologically, CPN involvement typically manifests as weakness in ankle dorsiflexion and eversion (foot drop) accompanied by sensory loss over the dorsum of the foot [6]. Electromyography (EMG) and nerve conduction studies (NCS) are useful to localize the lesion, quantify severity, and assess reinnervation potential. In the 2023 review by Mungalpara et al., ~71% of reported cases had EMG/NCS performed [4]. MRI is the gold standard imaging modality to characterize the cyst’s morphology, extent, and relationship to the nerve, as well as to confirm communication with the PTFJ (a communication was identified in ~91% of cases in that series) [4]. High-resolution ultrasound (HRUS) is a valuable adjunct, providing a rapid, dynamic assessment and guiding aspiration procedures [9,10].

Management options range from observation to surgical excision of the cyst with adjunctive joint procedures. According to Mungalpara et al. [4], recurrence rates after various treatments were 77% after aspiration (±steroid injection), 56% after simple excision, 11.5% after excision with resection of the recurrent articular branch of the CPN, and 0% after excision with PTFJ arthrodesis. Definitive surgery typically entails cyst excision combined with addressing the joint connection (e.g., ligation of the recurrent articular branch of the CPN or arthrodesis of the PTFJ) to minimize recurrence [11,12]. However, ultrasound-guided aspiration offers rapid symptom relief, is low-risk, and is especially useful for patients unwilling or unfit for surgery [9,10]. Indeed, image-guided aspiration of meniscal or PTFJ cysts has been described as early as 1992 and in subsequent case series with generally good short-term outcomes [13,14]. Early decompression of the nerve is crucial, as delays have been linked to irreversible nerve damage, specifically axonal loss and fibrosis [5,15].

Here, we report the case of a recreational long-distance runner who developed an acute-on-chronic CPN palsy due to an extraneural PTFJ ganglion cyst. Despite severe electrophysiological impairment, she achieved a rapid and near-complete motor recovery following ultrasound-guided cyst aspiration and rehabilitation. This case adds to the limited literature on such lesions in athletic populations and underscores the potential of image-guided aspiration as a temporizing or alternative treatment option in selected patients.

## Case presentation

A 36-year-old female recreational long-distance runner with no significant past medical history (no prior surgeries, medications, or allergies) and no relevant family history presented with a six-month history of progressive pain in the lateral aspect of the left knee. Initially, the knee pain was mechanical in nature, localized to the lateral joint area, and did not limit her training or participation in half-marathons. Approximately one month before presentation, she developed tingling and numbness (paresthesia) over the dorsum of the left foot, accompanied by progressively worsening ankle dorsiflexion weakness, to the point that she could no longer run safely. She denied any history of trauma, recent illness or surgery, or prolonged limb immobilization.

On physical examination, a visible and palpable swelling was noted on the lateral aspect of the left knee, just inferior to Gerdy’s tubercle, and it was tender to palpation. Neurological assessment revealed marked weakness in the muscles innervated by the CPN: dorsiflexors and evertors were significantly impaired, with Medical Research Council (MRC) grades of 2/5 for the tibialis anterior, 1/5 for the extensor hallucis longus, 1/5 for the extensor digitorum longus, and 1/5 for the fibularis (peroneal) muscles (Video 1). Sensation was decreased over the dorsal foot, most prominently in the first interdigital space. Gait examination showed a high-steppage pattern due to an obvious foot drop.

**Video 1:** Clinical evaluation before ganglion cyst aspiration.

Electrodiagnostic studies had been obtained prior to referral (Table 1). NCS showed normal sensory responses in the sural and superficial peroneal nerves, as well as normal motor conduction in the tibial nerve (including normal F-wave latencies). In the CPN, however, there was a >50% drop in compound motor action potential amplitude across the fibular head segment, with a slowed conduction velocity of 38 m/s at that site. These findings localized the lesion to the CPN at the fibular head (motor fibers), below the take-off of the superficial peroneal (sensory) branch, and above the branch to tibialis anterior. EMG demonstrated evidence of denervation in the tibialis anterior muscle with only scant signs of reinnervation, while other muscles (e.g., vastus lateralis and biceps femoris) were normal.

**Table 1:** Summary of electromyography (EMG) and nerve conduction study (NCS) findings. CPN: common peroneal nerve

Test	Nerve/muscle	Result	Notes
Sensory NCS	Sural nerve	Normal	—
Sensory NCS	Superficial peroneal nerve	Normal	—
Motor NCS	Posterior tibial nerve	Normal (normal F-wave)	—
Motor NCS	Common peroneal nerve (fibular head segment)	>50% amplitude drop; slowed conduction velocity 38 m/s	Lesion localized at the fibular head (motor fibers)
EMG (needle exam)	Tibialis anterior (left)	Denervation potentials, few reinnervated motor units	Consistent with acute/chronic denervation (CPN motor branch)
EMG (needle exam)	Vastus lateralis, vastus medialis, biceps femoris, gastrocnemius medialis (bilateral)	Normal EMG recruitment	Innervated by femoral/sciatic branches (no proximal nerve lesion)

**Figure 1:**
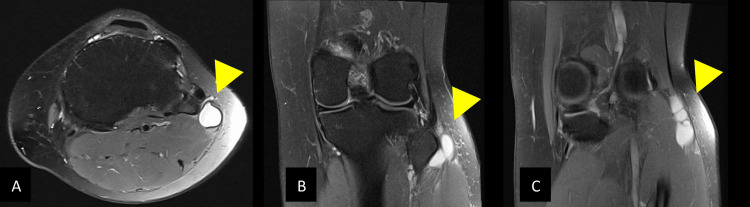
MRI of the left knee demonstrating a multiloculated cystic lesion consistent with a ganglion cyst arising from the proximal tibiofibular joint. (A) Axial T2-weighted fat-suppressed image at the level of the fibular head shows a well-defined, thin-walled, hyperintense ganglion cyst. (B, C) Coronal T2-weighted fat-suppressed images depict the multiloculate cyst extending distally along the head and proximal fibular diaphysis and proximally toward the distal biceps femoris tendon. MRI: magnetic resonance imaging

MRI of the left knee revealed a lobulated, thin-walled, fluid-filled cystic lesion measuring approximately 6 cm in length and up to 1.1 cm in diameter (Figure 1). It was located adjacent to the proximal fibula, extending distally along the upper fibular diaphysis (between the fibula and the peroneus longus muscle) and extending proximally toward the distal biceps femoris tendon. The lesion was in close contact with the distal CPN, although there was no evidence of intrinsic signal change within the nerve or acute muscle edema to suggest intraneural involvement. The lateral meniscus and major ligaments of the knee were intact. An incidental focal grade III chondropathy (~3 × 3 mm) of the medial patellar facet was noted.

In our rehabilitation intervention unit, a HRUS confirmed a multiloculated cyst with anechoic content in close relation to the CPN at the fibular head. Given the absence of trauma, the recent rapid progression of neurological deficits, and the patient’s preference to avoid surgery, we proceeded with ultrasound-guided percutaneous aspiration of the cyst as a minimally invasive decompression strategy. This was followed by an outpatient rehabilitation program for strengthening and gait training.

Initial intervention

**Figure 2:**
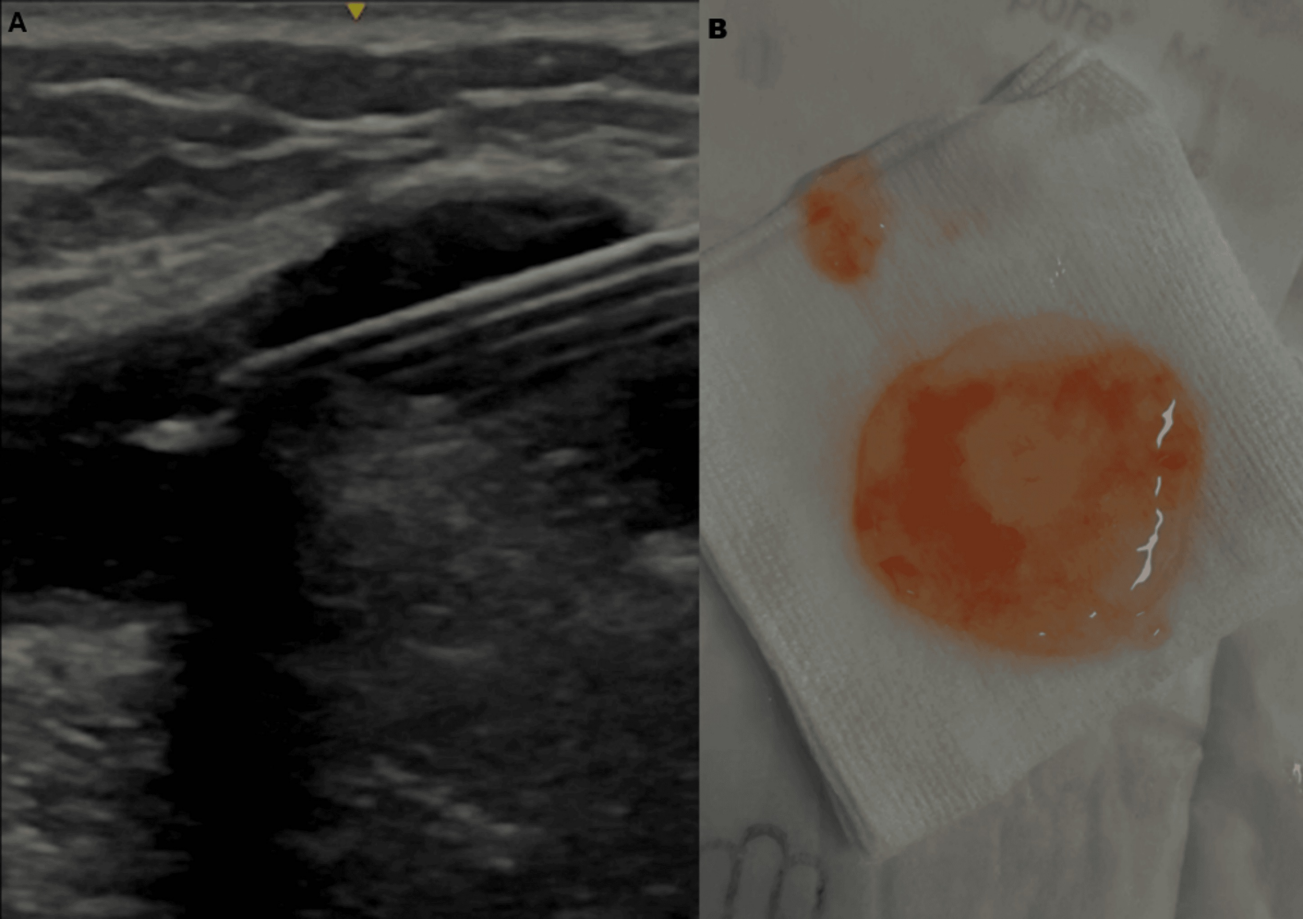
Ultrasound-guided aspiration of a proximal tibiofibular joint ganglion cyst (A) showing needle placement within the lesion, and gross appearance of the aspirated mucinous material with viscous gelatinous content and mild hematic admixture (B).

Under aseptic conditions and after obtaining written informed consent, the patient underwent ultrasound-guided percutaneous cyst aspiration. Using a high-frequency linear transducer (14 MHz), the multiloculated cyst was identified adjacent to the CPN at the fibular head. Local anesthesia was achieved with 1% lidocaine, and a 16-gauge needle was advanced into the cyst under real-time ultrasound guidance (Figure 2A). Approximately 2-3 mL of thick gelatinous fluid was aspirated from multiple locules, followed by saline lavage of the cyst cavity (Figure 2B). We then injected 40 mg of methylprednisolone acetate (Depo-Medrol^®^) mixed with 1 mL of 0.5% ropivacaine into the cyst. No immediate complications occurred. The patient was advised to resume activities as tolerated, to use an ankle-foot orthosis (AFO), and to begin a physiotherapy program. Given the severity of dorsiflexion weakness and foot drop, the use of an AFO was recommended to enhance ankle stability, improve gait safety, and reduce the risk of falls. However, the patient declined due to concerns about its appearance and because she did not perceive a personal need for it. Instead, she adopted a compensatory high-hip-flexion gait pattern on the affected side to clear the foot during the swing phase. The patient subsequently underwent a structured outpatient rehabilitation program at an external clinic. Although the specific session protocols were not available for detailed reporting, the program included progressive strengthening of ankle dorsiflexors and evertors, functional electrical stimulation of the dorsiflexors, proprioceptive training, gait re-education, and functional conditioning.

One-month follow-up

One month after the aspiration, the patient noted dramatic improvement. Motor strength in the affected muscles had improved to MRC 4/5 or better, and the foot drop had resolved, allowing a normal gait without the need for orthosis. She had minimal knee pain at this stage and was continuing with physiotherapy.

Two-month follow-up (second intervention)

By two months post-aspiration, the patient reported near-complete resolution of knee pain and further improvement in sensation. Her gait was normal. Manual muscle testing showed that strength had nearly returned to full: tibialis anterior 5/5, extensor hallucis longus 4/5, extensor digitorum longus 5/5, and fibularis muscles 5/5. However, mild numbness and tingling persisted over the lateral dorsum of the foot. To address these residual sensory symptoms, a second image-guided intervention was performed. Under ultrasound guidance, we administered a perineural injection of 4 mg of dexamethasone around the CPN (at the level of the sciatic bifurcation and proximal fibular tunnel). This steroid injection was intended to reduce any nerve irritation and improve the remaining paresthesia.

Three-month follow-up

At three months, the patient had achieved full motor recovery, with all previously weakened muscle groups now grading 5/5 on the MRC scale (Video 2). Only a mild hypoesthesia and dysesthesia persisted over the dorsum of the left foot (notably in the first interdigital space). The patient reported a recurrence of swelling and mild discomfort at the fibular head. Point-of-care ultrasound revealed a recurrent ganglion cyst at the proximal fibula. After discussing management options, the patient was referred to orthopedic surgery for evaluation and potential definitive surgical excision of the cyst. She was advised to continue her rehabilitation program in the meantime.

**Video 2:** Clinical evaluation at three-month follow-up.

Six-month follow-up (third intervention)

At six months, surgical removal of the cyst was still under consideration (the patient was deliberating between surgical and continued conservative management). Functionally, she remained independent and had even resumed running short distances without instability or foot drop. Her only complaint was a mild residual numbness on the dorsum of the foot, which was noticeable mainly when wearing tight-fitting shoes. By this time, she had completed a total of 24 physiotherapy sessions (approximately 2-3 sessions per week), which included electrostimulation of the anterior tibial muscle, progressive resistance exercises for the lower limb, cycling for endurance, and proprioceptive/balance training. Given the cyst’s recurrence and the persistent sensory annoyance, a third image-guided procedure was performed for symptomatic relief. After informed consent, we conducted another ultrasound-guided aspiration and lavage of the recurrent peroneal cyst, followed by an intracystic injection of 8 mg of dexamethasone. The procedure was well tolerated with no complications. The patient continued with maintenance physiotherapy while awaiting the orthopedic consultation for possible surgical intervention. The patient’s clinical course underscores the potential of ultrasound-guided aspiration as a minimally invasive temporizing option. Table 2 provides an overview of the patient’s clinical evolution and the therapeutic interventions undertaken.

**Table 2:** Timeline of clinical course, investigations, and interventions. MRI: magnetic resonance imaging; MRC: Medical Research Council; EHL: extensor hallucis longus; PTFJ: proximal tibiofibular joint; CPN: common peroneal nerve; TA: tibialis anterior; EDL: extensor digitorum longus

Time since initial visit	Clinical status and key findings	Interventions/management
Six months prior	Onset of lateral left knee pain (mechanical in nature, no functional limitation).	—
One month prior	Progression of symptoms: dorsal foot paresthesia and progressive ankle dorsiflexion weakness (incipient foot drop). EMG/NCS showed >50% conduction block at fibular head (38 m/s). MRI revealed a multiloculated 6 × 1.1 cm PTFJ ganglion cyst compressing the CPN.	—
Initial presentation (Day 0)	Foot drop with severe weakness (MRC: TA 2/5, EHL 1/5, EDL 1/5, fibularis 1/5); sensory loss in first interdigital space; palpable lateral knee swelling.	1st intervention: ultrasound-guided cyst aspiration and lavage; intracystic injection of 40 mg methylprednisolone + 1 mL ropivacaine. Outpatient physiotherapy was initiated.
One month post-aspiration	Motor function improved markedly (foot drop resolved; MRC grades ~4–5/5 at ankle). Pain reduced significantly.	Physiotherapy program was continued.
Two months post-aspiration	Pain relief was nearly complete; mild residual dorsal foot numbness persisted. Strength returned fully except for EHL (MRC 4/5).	2nd intervention: ultrasound-guided perineural steroid injection (4 mg dexamethasone) at sciatic bifurcation/fibular head to address sensory symptoms.
Three months post-aspiration	Full motor recovery was achieved (MRC 5/5 all muscles). Mild dorsal foot hypoesthesia/dysesthesia persisted. Recurrence of fibular head swelling/discomfort was noted. Point-of-care ultrasound showed a recurrent ganglion cyst.	Patient was referred for orthopedic surgical evaluation. Physiotherapy was continued.
Six months post-aspiration	Normal motor function was sustained (no foot drop; running resumed). Mild persistent dorsal foot paresthesia remained, especially with tight shoes.	3rd intervention: ultrasound-guided cyst re-aspiration and lavage with intracystic 8 mg dexamethasone injection. Ongoing physiotherapy; surgical excision was planned.

Patient perspective

“I wanted to avoid surgery and return to running safely. The aspiration relieved my foot drop quickly, which was reassuring. I still notice some sensory changes between my big toe and second toe, especially when wearing tight shoes, but I’m satisfied with the function and the ability to train again.”

## Discussion

PTFJ ganglion cysts are an uncommon yet well-documented cause of CPN compression, with clinical presentations ranging from subtle sensory disturbance to complete foot drop. In the largest aggregated review (61 case reports and seven case series), Mungalpara et al. found that these cysts were intraneural in ~39% of cases, extraneural in ~26%, combined in ~15%, and intraosseous in ~4% [4]. Intraneural cysts track within the nerve via the articular branch, whereas extraneural cysts arise outside the nerve and compress it externally [7,16].

Extraneural cysts are more often palpable on exam and may be detected earlier than intraneural ones, since they occupy more superficial tissue planes [5]. Although both types can produce motor and sensory deficits, extraneural cysts tend to cause purely extrinsic compression rather than intraneural invasion. This distinction may partly explain the favorable prognosis for motor recovery when decompression is achieved early. In our patient, this principle was evident: despite presenting with an acute foot drop, severe conduction block on EMG, and rapid neurological decline, she experienced full restoration of motor function within one month of decompression.

Early recognition and intervention are critical, as prolonged nerve compression can lead to perineural edema, segmental demyelination, and ultimately Wallerian degeneration with irreversible fibrosis. Advanced axonal injury has been associated with poorer outcomes, underscoring the importance of timely decompression [5,15]. Imaging plays a central role in diagnosis and management. MRI is excellent for delineating cyst anatomy and its relationship to surrounding structures [4]. HRUS complements MRI by allowing dynamic examination and facilitating guided aspiration procedures [9,10]. In our case, ultrasound not only confirmed the extraneural multiloculated cyst seen on MRI but was also invaluable intraprocedurally for precise needle placement during aspiration and steroid injection.

Ultrasound-guided aspiration (often with steroid instillation) has been reported as a minimally invasive treatment for PTFJ ganglion cysts for several decades. Multiple small series have documented rapid symptomatic relief and functional improvement with this approach [9,10,14]. Our patient’s outcome aligns with these reports, as she had a rapid recovery of muscle strength and function following aspiration and rehabilitation. Importantly, however, the risk of cyst recurrence after aspiration alone is high. Published recurrence rates range widely, from about 13% to as high as 77%, depending on the patient population and techniques used [4,11]. In our patient, the cyst recurred within three months of the initial aspiration, which is consistent with this tendency for recurrence. Fortunately, her neurological function remained intact after a repeat aspiration, with only mild sensory symptoms. We suspect that the early recurrence in this case was related to the cyst’s large initial size, multiloculated nature, and persistent communication with the PTFJ.

From a pathophysiological perspective, failure to eliminate the cyst’s connection to the joint (particularly the articular branch in intraneural cysts) is a major contributor to recurrence [7,16]. In extraneural cysts as well, simply excising or aspirating the cyst without addressing the PTFJ connection leaves the underlying cause (synovial fluid tracking) unchecked, leading to a high likelihood of recurrence [7]. Surgical management aims to remove the cyst and disconnect it from the joint. Surgical excision combined with ligation or resection of the recurrent articular branch of the CPN - and in select cases, arthrodesis of the PTFJ - has been shown to yield the lowest recurrence rates (0% recurrence with excision plus arthrodesis) [12]. Even in cases of recurrence or delayed presentation, surgical intervention can achieve excellent outcomes if meticulous technique is used [17,18].

In summary, our case demonstrates that ultrasound-guided aspiration of an extraneural PTFJ ganglion cyst, combined with a structured rehabilitation program, can result in rapid and substantial neurologic recovery - even when there is severe initial nerve compression with conduction block and denervation. This minimally invasive approach can be an effective first-line or temporizing treatment, particularly for patients who are poor surgical candidates or who desire to avoid surgery. Nonetheless, given the high recurrence rate associated with aspiration alone, current evidence and expert consensus support surgical excision of the cyst with concurrent joint disconnection as the definitive long-term management strategy. This is especially pertinent for active patients (such as athletes) and for cases with recurrent or persistent cysts. In such patients, referral for orthopedic or neurosurgical evaluation is recommended to discuss definitive treatment options [4,5].

## Conclusions

This case illustrates that a PTFJ ganglion cyst, although rare, should be included in the differential diagnosis of foot drop in athletes, even in the absence of trauma. Ultrasound-guided aspiration combined with rehabilitation can achieve rapid and significant motor recovery in CPN palsy, even in cases with severe neurologic impairment at presentation, and may help prevent permanent deficits if performed early.

However, recurrence after aspiration is frequent, particularly if the cyst’s articular connection to the joint is not addressed. For long-term cure and prevention of recurrence, surgical excision of the ganglion cyst with disconnection of the PTFJ remains the gold standard, especially in active patients and those with recurrent lesions.
